# Factors Related to Receipt of Help for Alcohol Use: Extending the Focus of Treatment to the Continuum of Unhealthy Alcohol Use

**DOI:** 10.1177/29768357241301990

**Published:** 2024-11-25

**Authors:** Lina Tieu, Nadereh Pourat, Elizabeth Bromley, Rajat Simhan, Roshan Bastani, Beth Glenn

**Affiliations:** 1 Department of Health Policy and Management, UCLA Fielding School of Public Health, Los Angeles, CA, USA; 2 Center for Healthcare Policy and Research, University of California, Davis, Sacramento, CA, USA; 3 UCLA Center for Health Policy Research, Los Angeles, CA, USA; 4 Department of Psychiatry and Biobehavioral Sciences, University of California, Los Angeles, Los Angeles, CA, USA; 5 SJ Health, French Camp, CA, USA; 6 Center for Cancer Prevention and Control Research and UCLA-Kaiser Permanente Center for Health Equity, Fielding School of Public Health, Jonsson Comprehensive Cancer Center, UCLA, Los Angeles, CA, USA

**Keywords:** Alcohol, alcohol treatment, unhealthy alcohol use, behavioral medicine, healthcare disparities

## Abstract

**Background:**

Unhealthy alcohol use is one of the leading preventable causes of mortality in the U.S. Despite evidence of the growing burden of alcohol-associated mortality and disease, treatment is severely underutilized. Prior literature has often focused on assessing treatment among patients with severe alcohol use.

**Objectives:**

Assess factors associated with uptake of treatment for alcohol use among a broad population of those regularly exceeding U.S. guidelines for alcohol use.

**Design:**

Cross-sectional study.

**Methods:**

Using data from the National Epidemiologic Survey on Alcohol and Related Conditions – Wave III (NESARC-III) collected April 2012 to June 2013, weighted descriptive statistics were used to describe the U.S. population who self-reported regularly exceeding U.S. guidelines for moderate alcohol use at least monthly. Weighted multivariable regression was used to assess the association of individual-level factors with receipt of help for unhealthy alcohol use.

**Results:**

In weighted analyses of 6467 NESARC-III participants, 17% of the U.S. population reported regular engagement in unhealthy alcohol use (76% use exceeding guidelines, 14% binge drinking, 11% heavy drinking) and were predominantly male (62%), below age 65 (93%), non-Hispanic White (65%), and had lower levels of education and income. Half (53%) met criteria for alcohol use disorder. Only 5% reported receipt of help for their alcohol use. Compared to non-Hispanic White individuals, non-Hispanic Asian/Native Hawaiian or Other Pacific Islander (odds ratio [OR] 0.40, 95% confidence interval [CI] 0.18-0.90) and non-Hispanic Black (OR 0.68, 95% CI 0.48-0.96) individuals were less likely to receive help for alcohol use. Factors associated with greater receipt of help included being older, educational attainment, Medicaid insurance, concomitant drug use, liver disease, acute healthcare utilization, and greater alcohol-related problems.

**Conclusion:**

Identification of the factors associated with receipt of alcohol-related treatment, including race and ethnicity, age, education, insurance, and drug use can inform interventions to increase treatment receipt.

## Introduction

Unhealthy alcohol use has been defined as a spectrum of alcohol use spanning from alcohol consumption that exceeds guidelines for moderate alcohol use to heavier drinking that is likely to be associated with higher severity consequences and alcohol use disorder (AUD), a condition in which one’s alcohol use has led to difficulty controlling drinking despite experiencing serious problems.^1,2^ While the health effects of chronic and severe use of alcohol are established, there is a growing body of research indicating that any level of alcohol use may raise the risk of health consequences, including numerous cancers and cardiovascular disease.^3,4^ There is evidence that rates of unhealthy alcohol use and its associated consequences in the United States (U.S.) are increasing and have been exacerbated by the COVID-19 pandemic.^5-8^ In addition, studies have found that rates of alcohol-related mortality during the start of the pandemic were more pronounced among racial and ethnic minoritized groups, particularly American Indian and Alaska Native, Black, and Latinx adults, as well as women and young adults.^9-11^

Although there is lack of consistency in definitions of unhealthy alcohol use in guidelines published by different agencies (eg, U.S. Preventive Services Task Force [USPSTF], Centers for Disease Control and Prevention, Substance Abuse and Mental Health Services Administration [SAMHSA], National Institute on Alcohol Abuse and Alcoholism [NIAAA]), classifications of alcohol use have defined different consumption patterns along a continuum of severity.^1^ Increasingly, evidence has supported guidelines and other efforts to address alcohol use within primary care settings to promote identification and initiation of treatment earlier in the continuum of unhealthy alcohol use, as opposed to after the experience of more severe consequences.^1,12^

Despite the increasing burden and consequences of unhealthy alcohol use, estimates have indicated that only 20% of individuals with alcohol dependence report ever receiving treatment.^13^ Among those with high severity use (eg, substance use disorder), studies have found that being older, married, having health insurance, and experiencing a greater number of problems are associated with receipt of treatment for substance use.^14-18^ In contrast, there is evidence that racial and ethnic minoritized groups are less likely to receive care for unhealthy alcohol use.^19^ Barriers to seeking treatment include lack of affordable care, attitudinal barriers (eg, alcohol-related problems will get better on their own), lower readiness for change, and stigma.^20-22^ There is evidence that among those with alcohol-related diagnoses, alcohol-related liver disease is negatively associated with receipt of pharmacotherapy.^23^ Additionally, while prior literature has noted the significance of comorbid use of alcohol and other substances on treatment outcomes, less is known about the relationship between substance use and specific treatment for alcohol.^24,25^

While prior studies have largely focused on assessing treatment uptake for alcohol use that has caused severe consequences, such as AUD or alcohol-related liver disease, there is a dearth of research on treatment across the broader continuum of unhealthy alcohol use.^26^ Among individuals reporting regular engagement in unhealthy alcohol use (ie, reporting monthly alcohol use that exceeds U.S. guidelines for drinking), this study sought to (1) Describe the characteristics of individuals who regularly engaged in unhealthy alcohol use, including how these characteristics differed between individuals with different patterns of drinking along the continuum of alcohol use, and (2) Assess the individual-level factors associated with receiving help for alcohol use.

## Methods

### Research Design

This cross-sectional study is a secondary data analysis using data from a population-based survey, the National Epidemiologic Survey on Alcohol and Related Conditions (NESARC-III).

### Data Source

NESARC-III, administered by NIAAA, sampled 36 309 non-institutionalized adults in the U.S from April 2012 to June 2013.^13^ The NESARC-III survey used multistage probability sampling, and validated responses among 17.4% of the final survey sample.^27,28^ Specific populations, including Hispanic/Latino, Black, Asian, and young (18-24) individuals, were oversampled.^13^ All NESARC-III participants provided written informed consent.^27^ While there are more recent surveys that assess alcohol use within the U.S. population, many focus on specific patterns of drinking (eg, binge drinking), specific timeframes (eg, drinking within the last month), or have more limited sample sizes or information on receipt of help for alcohol use.^29^ Thus, NESARC-III remains one of the most comprehensive population-based sources of data on alcohol use that would allow for the assessment of a broad continuum of unhealthy alcohol use, alcohol-related consequences, and receipt of treatment necessary for this analysis.^30^ Questions from the Alcohol Use Disorder and Associated Disabilities Interview Schedule-5 (AUDADIS-5), which has been validated to assess substance use according to the Diagnostic and Statistical Manual of Mental Disorders Fifth Edition (DSM-5) criteria within the general population, were used to assess alcohol consumption and experiences.^31^

### Conceptual Framework

**Figure 1.**
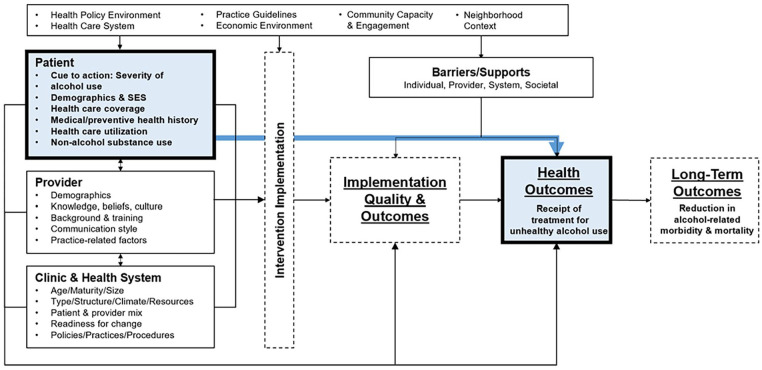
Multi-level health outcomes framework. Adapted from Bastani et al, UCLA, 1990-2022.^32-38^

The study used the Multi-Level Health Outcomes Framework (Figure 1), a multi-level, socioecological model that combines elements of conceptual frameworks related to the implementation of health interventions and delineates mutable (eg, health care coverage) and immutable factors (eg, demographic characteristics) at the individual/patient, provider, and health care setting levels that influence health behaviors and outcomes.^32-38^ This study adapted the framework to focus on the relationship between individual-level factors and receipt of treatment for unhealthy alcohol use. Specifically, immutable factors (eg, demographic characteristics) may influence the receipt of treatment for unhealthy alcohol use due to factors such as the availability of culturally-concordant treatment options and experiences of discrimination.^39^ Other patient-level factors included the severity of alcohol-related problems and medical conditions experienced as result of or exacerbated by alcohol use, which may influence patients’ and providers’ perceived need for treatment.^15^ Additionally, the relationship between mutable factors (eg, health insurance coverage, income) on receipt of treatment were assessed, as they may be addressed through specific interventions, such as policies to increase the affordability of care.^17,40^

### Sample and Inclusion Criteria

**Figure 2.**
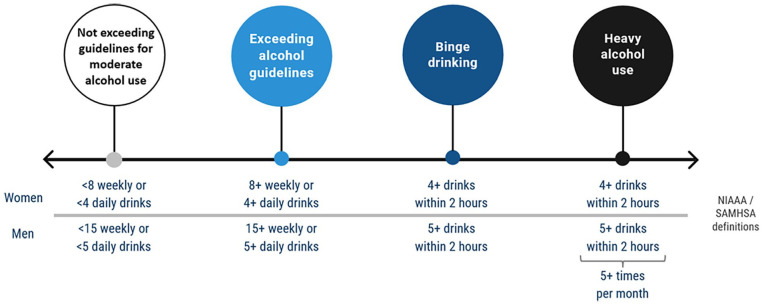
Continuum of alcohol use severity and definitions. Abbreviations: NIAAA, National Institute on Alcohol and Alcoholism; SAMHSA, Substance Abuse and Mental Health Services Administration.

The study sample included 6467 respondents of NESARC-III who reported regularly consuming alcohol (ie, at least monthly) at levels exceeding U.S. guidelines for moderate levels of alcohol use in the prior year, defined as consuming more than 7 drinks weekly or more than 3 daily drinks for women, and more than 14 weekly or more than 4 daily drinks for men.^41^ Following guidance from the USPSTF, individuals reporting exceeding these guidelines were defined as reporting *unhealthy alcohol use*, defined as alcohol use that either increases the risk of or has already led to health consequences, which are outlined by various agencies for categorizing patterns of risky alcohol use (Figure 2).^1^

### Outcome

The main outcome was receipt of help for alcohol use in the prior 12 months assessed via the question “Have you gone anywhere or seen anyone for a reason that was related in any way to your drinking?” Settings for receipt of help (not mutually exclusive) included: Alcoholics Anonymous or 12-step program, family services/social services agency, detox clinic, inpatient ward, outpatient clinic, rehab program, emergency department, halfway house/therapeutic community, crisis center, employee assistance program, religious leader, other health professional, other agency or professional.^42^

### Independent variables

The specification of independent variables is described in more detail in Supplemental Table 5.

#### Severity of unhealthy alcohol consumption

Using questions about frequency, type, and amount of alcohol consumption, NESARC calculates the number of standard drinks (containing 0.60 ounces of alcohol) consumed by participants.^42,43^ Guided by definitions of categories of unhealthy alcohol use from the NIAAA and SAMHSA, 3 mutually exclusive categories/patterns (from lowest to highest severity of alcohol use) of unhealthy alcohol use in the prior 12 months were specified: (1) Exceeding guidelines, defined as exceeding moderate levels of alcohol use (>7 weekly or >3 daily drinks for women, >14 weekly or >4 daily drinks for men) at least monthly, but not falling within binge drinking or heavy alcohol use categories, (2) Binge drinking, defined as consuming at least 4 (for women) or 5 (for men) drinks within 2 hours at least monthly, but not meeting criteria for heavy alcohol use, and (3) Heavy alcohol use, defined as binge drinking on 5 or more days a month at least monthly.^41,44,45^

#### Alcohol-related problems and alcohol use disorder

We followed DSM-5 criteria and definitions to classify alcohol-related problems and AUD severity (Supplemental Table 6). Binary variables (experienced vs did not experience) were constructed to reflect experience of each of 11 alcohol-related problems in the past year.^46^ Alcohol-related problems were defined as the sum (0-11) of alcohol-related problems reported in the past year.^46^ AUD severity was categorized as: no AUD (0-1 problems), mild AUD (2-3 problems), moderate AUD (4-5 problems), or severe AUD (6 or more problems).^46,47^ Due to its clinical significance but close relationship with total number of alcohol-related problems, AUD severity was assessed in descriptive analyses, but omitted from multivariable analyses.

### Covariates

#### Demographic variables

Age was categorized as being 18 to 34, 35 to 64, or 65 years or older. Sex was categorized as male or female. Educational attainment was defined as the highest grade level achieved, categorized as: (1) less than high school, (2) high school or General Educational Development (GED), (3) some college, (4) college or associate degree, or (5) more than a college degree. Race and ethnicity was categorized as: (1) Hispanic, any race; (2) Black, non-Hispanic; (3) White, non-Hispanic; (4) Asian/Native Hawaiian/Other Pacific Islander, non-Hispanic; and (5) American Indian/Alaska Native, non-Hispanic. Income was defined as annual household income in U.S. dollars, categorized as: (1) <20 000; (2) 20 000 to < 40 000; (3) 40 000 to < 70 000; (4) 70 000 to < 100 000; and (5) 100 000 or more. Nativity was categorized as being born in the U.S. versus outside of the U.S. Participants were categorized as having limited English proficiency if they noted speaking English poorly or very poorly, and proficient in English if they noted speaking English well, very well, or as their only language.

#### Health insurance

We categorized participants into 4 mutually-exclusive categories of health insurance coverage in the prior 12 months: (1) no insurance, (2) any Medicaid, (3) any private insurance, non-Medicaid, and (4) other insurance. Individuals were only categorized as having other insurance if they did not note having any Medicaid or Private Insurance coverage.

#### Medical conditions and healthcare utilization in prior 12 months

Medical conditions associated with unhealthy alcohol use included report of being diagnosed with (1) liver disease (including cancer) or (2) non-liver cancer. Acute health care utilization was defined as reporting an emergency department visit or hospitalization versus experiencing neither.

#### Substance use

To assess regular non-alcohol substance use, we assessed self-reported use of the following substances at least monthly: (1) marijuana, and (2) any non-alcohol substance except marijuana, including sedatives, painkillers, cocaine, stimulants, club drugs, hallucinogens, inhalants, and heroin.

Missing data in the NESARC-III for age, educational attainment, and household income were imputed by NIAAA based on other demographic variables.^27^ Participants missing data for the following variables were excluded from multivariable analyses: health insurance (n = 149), English proficiency (n = 88), nativity (n = 3), liver disease (n = 50), non-liver cancer (n = 42), acute healthcare utilization (n = 41), marijuana use (n = 6), and non-marijuana substance use (n = 8).

### Data analysis and weighting

Analyses were conducted using Stata 16 (College Station, TX). Descriptive statistics were used to compare individual-level characteristics and factors, stratified by alcohol use pattern and receipt of help for alcohol use. Bivariate analyses were used to assess unadjusted associations between individual-level factors and receipt of help for unhealthy alcohol use as they may be observed in applied settings.

A multivariable logistic regression was used to explore the relationship between demographic characteristics and individual-level factors associated with receipt of help for unhealthy alcohol use. To examine the goodness of fit of unweighted models, analyses applied and examined pseudo-*R*^2^, the Hosmer-Lemeshow Test, and the post-estimation Stata commands *linktest* and *fitstat.* Correlations were examined between all independent variables. Multicollinearity between independent variables was assessed using the Stata command *collin*.

#### Survey design effects

Population weighting was used to account for the complex survey design, survey non-response, and produce estimates representative of the U.S. noninstitutionalized adult population of 2012 (population size 235 411 957).^48^ The variables used in this analysis to account for sample stratification, clustering, and sample weights were produced by NIAAA.^27^

### Ethical codes and permissions

This analysis was prepared using a limited access dataset obtained through a data use agreement with NIAAA. This study was approved by the Institutional Review Board at University of California, Los Angeles (20-001322) and University of California, Davis (2 161 947-1).

## Results

### Description of sample

Overall, 6467 NESARC participants reported regular engagement in unhealthy alcohol use. Unweighted analyses are presented in Supplemental Tables 1 to 4. In weighted analyses, 40 210 509 (17% of the 2012 U.S. population) reported regular engagement in unhealthy alcohol use in the prior year. Within this sample, 76% regularly exceeded guidelines without meeting criteria for binge drinking/heavy alcohol use, 14% met criteria for binge drinking, and 11% met criteria for heavy drinking. Half met criteria for AUD (53%; Table 1).

**Table 1.** Alcohol Consumption and Experiences Among Adults with Past Year Unhealthy Alcohol Use, by Drinking Pattern, Weighted Sample (n = 40 210 509).

Characteristic, n (col %)	Exceeding guidelines	Binge drinking	Heavy alcohol use	Total
	N = 30 531 605 (76%)	N = 5 437 109 (14%)	N = 4 241 796 (11%)	N = 40 210 509
Sex				
Female	12 126 676 (40)	1 831 797 (34)	1 427 876 (34)	15 386 350 (38)
Male	18 404 929 (60)	3 605 311 (66)	2 813 919 (66)	24 824 159 (62)
Age				
18-34	13 240 597 (43)	3 175 239 (58)	1 985 336 (47)	18 401 173 (46)
35-64	14 760 995 (48)	2 117 096 (39)	2 145 340 (51)	19 023 431 (47)
65 or over	2 530 012 (8.3)	144 774 (2.7)	111 119 (2.6)	2 785 905 (6.9)
Race/Ethnicity				
Hispanic, any race	4 756 039 (16)	1 136 521 (21)	602 140 (14)	6 494 700 (16)
Black, non-Hispanic	3 977 575 (13)	630 946 (12)	850 107 (20)	5 458 628 (14)
White, non-Hispanic	20 157 114 (66)	3 341 194 (61)	2 523 121 (59)	26 021 428 (65)
Asian/Native Hawaiian/Other Pacific Islander, non-Hispanic	1 055 985 (3.5)	191 878 (3.5)	142 685 (3.4)	1 390 548 (3.5)
American Indian/Alaska Native, non-Hispanic	584 893 (1.9)	136 571 (2.5)	123 743 (2.9)	845 206 (2.1)
Education				
Less than high school	3 455 490 (11)	826 562 (15)	844 568 (20)	5 126 620 (13)
High school or GED	8 395 373 (27)	1 434 450 (26)	1 608 234 (38)	11 438 056 (28)
Some college	7 786 380 (26)	1 613 347 (30)	918 152 (22)	10 317 879 (26)
College or associate degree	7 470 859 (24)	1 247 583 (23)	725 889 (17)	9 444 332 (23)
More than college	3 423 502 (11)	315 167 (5.8)	144 953 (3.4)	3 883 623 (9.7)
Nativity				
Born in United States	27 157 145 (89)	4 753 753 (87)	3 970 099 (94)	35 880 998 (89)
Not born in United States	3 350 043 (11)	683 355 (13)	271 697 (6.4)	4 305 095 (11)
English Proficiency				
Speaks English well or very well	29 098 114 (95)	5 170 114 (95)	4 101 518 (97)	38 369 746 (95)
Speaks English poorly or very poorly	923 708 (3)	208 444 (3.8)	61 792 (1.5)	1 193 944 (3)
Income				
<$20 000	6 197 242 (20)	1 486 010 (27)	1 380 921 (33)	9 064 174 (23)
$20 000 to <40 000	6 747 141 (22)	1 374 782 (25)	1 135 288 (27)	9 257 211 (23)
$40 000 to <70 000	7 196 457 (24)	1 218 784 (22)	995 937 (23)	9 411 177 (23)
$70 000 to <100 000	4 383 342 (14)	596 798 (11)	402 467 (9.5)	5 382 606 (13)
$100 000 or more	6 007 423 (2)	760 735 (14)	327 184 (7.7)	7 095 341 (18)
Insurance status				
No insurance	6 849 710 (22)	1 561 320 (29)	1 376 345 (32)	9 787 376 (24)
Any medicaid	3 033 319 (9.9)	580 318 (11)	578 256 (14)	4 191 893 (10)
Any private, non-medicaid	16 729 524 (55)	2 748 956 (51)	1 706 769 (40)	21 185 248 (53)
Other insurance, non-medicaid, non-private	3 266 792 (11)	409 537 (7.5)	447 432 (11)	4 123 761 (10)
Medical conditions, Last 12 mo				
Any liver disease (cirrhosis, liver cancer, other liver disease)	418 367 (1.4)	32 540 (0.6)	150 834 (3.6)	601 741 (1.5)
Any non-liver cancer	796 406 (2.6)	66 852 (1.2)	73 952 (1.7)	937 210 (2.3)
Acute healthcare utilization (ED visit or hospitalization), last 12 mo	7 025 822 (23)	1 403 568 (26)	1 191 456 (28)	9 620 846 (24)
Regular non-alcohol substance use, last 12 mo				
Marijuana	4 804 596 (16)	1 477 884 (27)	1 342 254 (32)	7 624 734 (19)
Any non-Marijuana substance	2 078 965 (6.8)	576 536 (11)	774 147 (18)	3 429 648 (8.5)
Alcohol use disorder, last 12 mo	14 554 729 (48)	3 489 911 (64)	3 372 922 (80)	21 417 562 (53)
None (0-1 alcohol-related problems)	15 991 577 (52)	1 956 034 (36)	872 795 (21)	18 820 406 (47)
Mild (2-3 alcohol-related problems)	7 061 455 (23)	1 241 483 (23)	634 751 (15)	8 937 690 (22)
Moderate (4-5 alcohol-related problems)	3 869 410 (13)	947 053 (17)	746 001 (18)	5 562 464 (14)
Severe (⩾6 alcohol-related problems)	3 609 163 (12)	1 292 538 (24)	1 988 249 (47)	6 889 950 (17)
Received help for alcohol use, last 12 mo				
Sought help for drinking (from any source)	1 136 980 (3.7)	348 119 (6.4)	681 630 (16)	2 166 729 (5.4)
Perceived need, but did not seek help	812 468 (2.7)	220 598 (4.1)	359 065 (8.5)	1 392 131 (3.5)
Did not perceive need for or seek help	28 582 157 (94)	4 868 392 (90)	3 201 100 (75)	36 651 649 (91)
Source of help for alcohol use, last 12 mo				
Alcoholics/narcotics/cocaine anonymous or 12-step meeting	678 435 (2.2)	183 411 (3.4)	422 504 (10.0)	1 284 350 (3.2)
Private physician, psychiatrist, psychologist, social worker, other professional	491 973 (1.6)	161 902 (3.0)	316 690 (7.5)	970 565 (2.4)
Outpatient clinic, including outreach and day/partial patient program	300 106 (1.0)	117 504 (2.2)	190 240 (4.5)	607 849 (1.5)
Alcohol/drug rehabilitation program	242 339 (0.8)	93 793 (1.7)	130 885 (3.1)	467 017 (1.2)
Alcohol/drug detoxification ward/clinic	155 342 (0.5)	66 209 (1.2)	166 633 (3.9)	388 184 (1.0)
Emergency room	169 216 (0.6)	45 037 (0.8)	186 671 (4.4)	400 924 (1.0)
Family services or other social services agency	179 046 (0.6)	68 817 (1.3)	111 337 (2.6)	359 200 (0.9)
Clergyman, priest, or rabbi	134 716 (0.4)	33 644 (0.6)	93 232 (2.2)	261 593 (0.7)
Inpatient alcohol/drug ward/clinic	98 954 (0.3)	43 516 (0.8)	129 336 (3.0)	271 806 (0.7)
Halfway house/therapeutic community	52 808 (0.2)	16 280 (0.3)	40 495 (1.0)	109 583 (0.3)
Crisis center	10 510 (0.03)	2779 (0.1)	36 869 (0.9)	50 159 (0.1)
Employee assistance program	48 039 (0.2)	5345 (0.1)	8776 (0.2)	62 161 (0.2)
Other agency or professional	61 358 (0.2)	10 767 (0.2)	20 799 (0.5)	92 923 (0.2)

Abbreviations: ED, emergency department; GED, general educational development.

The majority of the weighted sample reporting regular engagement in unhealthy alcohol use were male (62%); below age 65 (93%); Non-Hispanic White (65%); had less than a college degree education (70%); and had an annual household income of less than $70,000 (69%). Most were born in the United States (89%) and had spoken English proficiency (95%). Half (53%) had private health insurance, a quarter (24%) had no health insurance, and 10% had Medicaid. While few reported severe alcohol-related medical conditions, almost one quarter (24%) reported past year acute health care utilization. A minority reported regular use of marijuana (19%) or other non-alcohol substance (9%).

In weighted analyses, few (5%) received help for alcohol use. The most common sources of help were 12-step programs (3%), private practice providers (2%), and outpatient clinics (2%). The proportion of individuals who received help for alcohol use increased with the severity of alcohol use (from 4% of those who exceeded guidelines to 16% of those with heavy alcohol use).

### Factors related to receipt of help for alcohol use

Among the weighted sample, receipt of help was most common among individuals aged 35 to 64 (6%); individuals reporting American Indian/Alaska Native, non-Hispanic race and ethnicity (11%); those born in the United States (6%); individuals with less than $20,000 annual household income; and Medicaid enrollees (10%; Table 2). Almost half (46%) of individuals with confirmed liver disease in the prior year reported receiving help for unhealthy alcohol use. Among individuals with AUD, one-fifth (21%) with severe AUD received help for unhealthy alcohol use.

**Table 2.** Alcohol Consumption and Experiences among Adults with Past Year Unhealthy Alcohol Use, by Treatment Utilization, Weighted Sample (n = 40 210 509).

Characteristic, n (row %)	Did not receive help	Received help	Total who exceeded guidelines	*P*-value
	N = 36 651 649	N = 2 166 729	N = 40 210 509	
Sex				.254
Female	14 622 347 (95.0)	764 003 (5.0)	15 386 350	
Male	23 421 433 (94.0)	1 402 726 (5.7)	24 824 159	
Age				<.001
18-34	17 464 152 (95)	937 021 (5.1)	18 401 173	
35-64	17 824 851 (94)	1 198 580 (6.3)	19 023 431	
65 or over	2 754 777 (99)	31 128 (1.1)	2 785 905	
Race/Ethnicity				.024
Hispanic, any race	6 188 408 (95.0)	306 292 (4.7)	5 126 620	
Black, non-Hispanic	5 157 565 (94.0)	301 063 (5.5)	11 438 056	
White, non-Hispanic	24 587 155 (94.0)	1 434 274 (5.5)	10 317 879	
Asian/Native Hawaiian/Other Pacific Islander, non-Hispanic	1 355 594 (97.0)	34 953 (2.5)	9 444 332	
American Indian/Alaska Native, non-Hispanic	755 058 (89.0)	90 148 (11.0)	3 883 623	
Education				.063
Less than high school	4 824 395 (94.0)	302 226 (5.9)	5 126 620	
High school or GED	10 719 323 (94.0)	718 734 (6.3)	11 438 056	
Some college	9 700 160 (94.0)	617 718 (6.0)	10 317 879	
College or associate degree	9 073 113 (96.0)	371 219 (3.9)	9 444 332	
More than college	3 726 790 (96.0)	156 833 (4.0)	3 883 623	
Nativity				.012
Born in United States	33 830 554 (94.0)	2 050 444 (5.7)	35 880 998	
Not born in United States	4 188 810 (97.0)	116 285 (2.7)	4 305 095	
English Proficiency				
Speaks English well or very well	36 264 356 (95)	2 105 390 (5.5)	38 369 746	.251
Speaks English poorly or very poorly	1 160 689 (97)	33 255 (2.8)	1 193 944	
Income				<.001
<$20 000	8 201 465 (90)	862 709 (9.5)	9 064 174	
$20 000 to <40 000	8 730 797 (94)	526 414 (5.7)	9 257 211	
$40 000 to <70 000	9 016 090 (96)	395 088 (4.2)	9 411 177	
$70 000 to 100 000	5 163 130 (96)	219 476 (4.1)	5 382 606	
$100 000 or more	6 932 298 (98)	163 043 (2.3)	7 095 341	
Insurance status				<.001
No insurance	9 137 600 (93)	649 776 (6.6)	9 787 376	
Any medicaid	3 699 139 (88)	492 754 (10.2)	4 191 893	
Any private, non-medicaid	20 523 064 (97)	662 184 (3.1)	21 185 248	
Other insurance, non-medicaid, non-private	3 833 959 (93)	289 802 (7.0)	4 123 761	
Medical conditions, last 12 mo				
Any liver disease (cirrhosis, liver cancer, other liver disease)	326 285 (54)	275 456 (46.0)	601 741	<.001
Any non-liver cancer	867 939 (93)	69 271 (7.4)	937 210	.227
Acute healthcare utilization (ED visit or hospitalization), last 12 mo	9 451 356 (91)	990 821 (9.5)	10 442 177	<.001
Regular non-alcohol substance use, last 12 mo				
Marijuana	6 948 300 (91)	676 435 (8.9)	7 624 734	<.001
Any non-Marijuana substance	2 870 315 (84)	559 332 (16.0)	3 429 648	<.001
Alcohol use disorder, last 12 mo				<.001
None (0-1 alcohol-related problems)	18 623 273 (99)	197 133 (1)	18 820 406	
Mild (2-3 alcohol-related problems)	8 709 018 (97)	228 672 (2.6)	8 937 690	
Moderate (4-5 alcohol-related problems)	5 280 076 (95)	282 388 (5.1)	5 562 464	
Severe (⩾6 alcohol-related problems)	5 431 413 (79)	1 458 537 (21)	6 889 950	

Abbreviations: ED, emergency department; GED, general educational development.

In weighted bivariate results (Table 3), factors associated with receipt of help included higher severity of unhealthy alcohol use, American Indian/Alaska Native, non-Hispanic race and ethnicity (vs. White, non-Hispanic), Medicaid enrollment (vs. no health insurance), having an alcohol-related medical condition, acute healthcare utilization, regular use of a non-alcohol substance, and greater number of alcohol-related problems. Factors associated with lower receipt of help included higher income, being born outside of the U.S., and having private health insurance.

**Table 3.** Bivariate Results, Weighted Sample.

Characteristic, OR	Did not receive help	Received help	*P*-value
	N = 36 651 649	N = 2 166 729	
Unhealthy alcohol use, past 12 mo			<.001
Exceeding guidelines	Ref	ref	
Binge drinking	Ref	1.77 (1.21-2.59)	
Heavy alcohol use	Ref	4.95 (3.60-6.80)	
Age			.002
18-34	Ref	ref	
35-64	Ref	1.25 (0.96-1.64)	
65+	Ref	0.21 (0.89-0.50)	
Sex			.254
Male	Ref	ref	
Female	Ref	0.87 (0.69-1.10)	
Race/Ethnicity			.034
White, non-Hispanic	Ref	ref	
Hispanic, any race	Ref	0.85 (0.62-1.17)	
Black, non-Hispanic	Ref	1.00 (0.78-1.28)	
Asian/Native Hawaiian/Other Pacific Islander, non-Hispanic	Ref	0.44 (0.19-1.04)	
American Indian/Alaska Native, non-Hispanic	Ref	2.05 (1.03-4.08)	
Education			.044
Less than high school	Ref	ref	
High school or GED	Ref	1.07 (0.69-1.67)	
Some college	Ref	1.02 (0.67-1.54)	
College or associate degree	Ref	0.65 (0.41-1.04)	
More than college	Ref	0.67 (0.35-1.30)	
Income			<.001
<$20 000	Ref	ref	
$20 000 to <40 000	Ref	0.57 (0.43-0.77)	
$40 000 to <70 000	Ref	0.42 (0.30-0.59)	
$70 000 to 100 000	Ref	0.40 (0.26-0.62)	
$100 000 or more	Ref	0.22 (0.12-0.43)	
Nativity			.002
Born in United States	Ref	ref	
Not born in United States	Ref	0.46 (0.28-0.74)	
English Proficiency *assumes English-only speakers exempt			.071
Speaks English well or very well	Ref	ref	
Speaks English poorly or very poorly	Ref	0.50 (0.23-1.06)	
Insurance status			<.001
No insurance	Ref	ref	
Any medicaid	Ref	1.87 (1.31-2.68)	
Any private, non-medicaid	Ref	0.45 (0.32-0.64)	
Other insurance, non-medicaid, non-private	Ref	1.06 (0.68-1.67)	
Medical conditions, last 12 mo (ref: no respective disease)			
Any liver disease (cirrhosis, liver cancer, other liver disease)	Ref	16.98 (10.31-27.94)	<.001
Any non-liver cancer (including breast, oropharyngeal, other cancer)	Ref	1.42 (0.74-2.76)	.292
Acute healthcare utilization, last 12 mo (ref: no visit)	Ref	2.58 (2.04-3.25)	<.001
⩾ Monthly non-alcohol substance use, last 12 mo			
Marijuana	Ref	2.03 (1.52-2.70)	<.001
Any non-Marijuana substance	Ref	4.27 (3.02-6.03)	<.001
Number of alcohol-related problems, last 12 mo	Ref	1.53 (1.47-1.60)	<.001

Abbreviations: ED, emergency department; GED, general educational development.

Examination of correlation and multicollinearity revealed no indicators of collinearity between independent variables. In weighted multivariable analysis (Table 4), the sole factor found to be associated with lower likelihood of receiving help for unhealthy alcohol use was race and ethnicity. Compared to White, non-Hispanic individuals, individuals reporting Black, non-Hispanic race and ethnicity (adjusted odds ratio [aOR] 0.68, 95% confidence interval [CI] 0.48-0.96) or Asian/Native Hawaiian, Other Pacific Islander, non-Hispanic race and ethnicity (aOR 0.40, 95% CI 0.18-0.90) were less likely to report receiving help for alcohol use.

**Table 4.** Multivariable Results for Receipt of Help for Unhealthy Alcohol Use, Weighted Sample.

Characteristic	OR (95% CI)	*P*-value
Unhealthy alcohol use, past 12 mo		
Exceeding moderate alcohol use	Ref	
Binge drinking	1.03 (0.65-1.61)	.906
Heavy alcohol use	1.47 (0.98-2.19)	.061
Age		
18-34	Ref	
35-64	1.48 (1.08-2.03)	.016
65+	0.47 (0.15-1.47)	.193
Sex		
Male	Ref	
Female	0.78 (0.58-1.05)	.102
Race/Ethnicity		
White, non-Hispanic	Ref	
Hispanic, any race	0.85 (0.57-1.26)	.412
Black, non-Hispanic	0.68 (0.48-0.96)	.028
Asian/Native Hawaiian/Other Pacific Islander, non-Hispanic	0.40 (0.18-0.90)	.027
American Indian/Alaska Native, non-Hispanic	0.81 (0.33-1.99)	.643
Education		
Less than high school	Ref	
High school or GED	1.52 (0.82-2.84)	.182
Some college	1.72 (0.94-3.12)	.076
College or associate degree	1.56 (0.84-2.89)	.155
More than college	2.80 (1.20-6.52)	.018
Income		
<$20 000	Ref	
$20 000 to <40 000	0.84 (0.59-1.20)	.336
$40 000 to <70 000	0.78 (0.52-1.16)	.211
$70 000 to <100 000	1.01 (0.56-1.82)	.976
$100 000 or more	0.51 (0.25-1.01)	.052
Nativity		
Born in United States	Ref	
Not born in United States	0.58 (0.29-1.16)	.123
English proficiency		
Proficiency	Ref	
Limited proficiency	1.47 (0.52-4.15)	.465
Insurance status		
No insurance	Ref	
Any medicaid	2.08 (1.31-3.30)	.002
Any private, non-medicaid	0.64 (0.41-1.00)	.050
Other insurance, non-medicaid, non-private	1.36 (0.79-2.35)	.262
Medical conditions, last 12 mo (ref: no respective disease)		
Any liver disease (cirrhosis, liver cancer, other liver disease)	7.57 (3.80-15.05)	<.001
Any non-liver cancer (including breast, oropharyngeal, other cancer)	0.84 (0.34-2.05)	.702
Any acute healthcare utilization (ED or hospitalization), last 12 mo (ref: no visit)	1.56 (1.16-2.11)	.004
⩾ Monthly non-Marijuana substance use, last 12 mo (ref: <monthly use)	1.51 (0.99-2.31)	.055
⩾ Monthly Marijuana use, last 12 mo (ref: <monthly use)	0.89 (0.63-1.26)	.513
Number of alcohol-related problems, last 12 mo	1.46 (1.38-1.54)	<.001

Abbreviations: ED, emergency department; GED, general educational development.

In weighted multivariable analyses, older age (35-64 vs 18-34) was associated with a higher odds of receiving help for unhealthy alcohol use (aOR 1.48, 95% CI 1.08-2.03). Compared to individuals reporting less than high school education, those reporting more than a college education had a higher likelihood of receiving help (aOR 2.80, 95% CI 1.20-6.52). Having Medicaid (vs. no insurance) was associated with increased likelihood of receipt of help (aOR 2.08, 95% CI 1.31-3.30). Individuals with liver disease (aOR 7.57, 95% CI 3.80-15.05) or any acute healthcare utilization (aOR 1.54, 95% CI 1.20-1.99) had a higher likelihood of receiving help. Experience of an additional alcohol-related problem was associated with a 46% increase in the likelihood of receiving help for unhealthy alcohol use (aOR 1.46, 95% CI 1.38-1.54). Additionally, monthly non-marijuana substance use was associated with greater odds of receipt of help (aOR 1.43, 95% CI 1.04-1.95).

## Discussion

In a nationally-representative sample of U.S. adults, almost 1 in 5 reported regular engagement in unhealthy alcohol use in the prior year. Within this sample, a large proportion reported unhealthy alcohol use without meeting criteria for higher severity consumption patterns (ie, binge drinking, heavy alcohol use) or AUD. These results demonstrate that a focus on individuals with high severity consumption patterns and AUD may underestimate the number of individuals who could benefit from intervention, supporting a shift to intervention earlier in the continuum of alcohol use.

Among those regularly exceeding alcohol use guidelines, we found that very few reported receiving help for their drinking, despite reporting a high burden of alcohol-related consequences (eg, alcohol-related problems and conditions) and half meeting criteria for AUD. Previous studies including individuals reporting alcohol-related problems or alcohol use disorder have found that barriers to help-seeking, including structural and attitudinal barriers, are highly prevalent.^20,49-51^

Among individuals regularly exceeding alcohol use guidelines, indicators of long-term or advanced consequences (ie, liver disease, increasing number of alcohol-associated problems) were associated with receipt of help. Prior research has found an association between the experience of more problems and treatment for alcohol, and that treatment is often delayed.^15,49,52-54^ While the identification and recognition of advanced consequences of alcohol use can serve as a driver of treatment, promoting earlier identification of unhealthy alcohol use has the potential to prevent more serious health consequences.

We found that racial and ethnic minoritized groups in our sample, including individuals reporting non-Hispanic Black or non-Hispanic Asian, Native Hawaiian, and Pacific Islander race and ethnicity, were significantly less likely to receive help. This finding is consistent with prior research showing lower receipt of care for alcohol use (ie, brief intervention) and higher report of structural barriers to care among racial and ethnic minoritized groups, including Black or African American and Asian or Pacific Islander patients.^19,20^ Addressing healthcare discrimination, increasing the diversity of the behavioral health workforce, and supporting providers in building cultural and linguistic competency may help improve provider-patient relationships and reduce perceived stigma related to treatment for alcohol use among diverse populations.^55-57^ Studies have also highlighted the influence of gender and intersectionality on receipt of care for alcohol use, including the finding of lower receipt of care among Black men and women and Asian and Pacific Islander women, and complex relationships between race and gender on barriers to care.^19,20^ While our study did not find an association between gender and treatment receipt in weighted analyses, there was evidence of an association of lower receipt of help among female NESARC respondents in unweighted analyses.

Prior research has found that greater educational attainment is associated with lower rates of unhealthy alcohol use.^58,59^ Our study expands upon past work by highlighting an association between advanced (postgraduate) education and increased receipt of help for alcohol use. The finding that Medicaid coverage but not private insurance was associated with greater receipt of help suggests the value of efforts to promote access to a comprehensive set of benefits for substance use treatment for patients seeking care from safety net health care settings.^60^ While the Mental Health Parity and Addiction Equity Act and inclusion of substance use treatment as essential health benefits within the Affordable Care Act have promoted improvements in coverage of mental health and substance use widely, remaining gaps include variability in Medicaid coverage between states, more restrictive coverage of substance use treatment services within private insurance plans, and limitations in provider networks.^61-63^

Limitations of this study include the timeframe of data collection (2012-2013). Given the use of secondary data, we did not perform a sample size analysis, but utilized weighting methods to account for the sampling methods of NESARC-III.^27^ However, NESARC-III remains one of the most comprehensive surveys on alcohol utilization, treatment, and experiences, and there is a dearth of more current surveys that offer a more complete assessment of unhealthy alcohol use at the lower end of the continuum, with many focusing on the frequency of binge drinking or other high severity patterns of use. To identify a sample regularly engaging in unhealthy alcohol use, the study used a cutoff based on alcohol consumption exceeding U.S. guidelines for moderate alcohol use. Thus, the study did not include individuals who did not meet such criteria, but for whom receipt of help may be appropriate, such as former drinkers and drinkers not regularly exceeding alcohol use guidelines but experiencing consequences. We did not directly measure the receipt or quality of alcohol-related treatment reported by survey participants. Due to the low prevalence of receipt of help, the outcome combined all sources of help which limited the ability to assess factors related to specific sources of intervention. Due to the cross-sectional nature of the analysis, causal inferences cannot be drawn regarding the influence of patient-identified problems and experiences on treatment-seeking.

This study identified a substantial subgroup of individuals currently left out of treatment who may benefit from care to reduce their alcohol use. The findings underscore the importance of implementing alcohol screening measures that capture a full continuum of unhealthy alcohol use to enable early identification within the general population. For example, standardized screening tools that capture frequency and amount of alcohol consumption (eg, Alcohol Use Disorders Identification Test [AUDIT]) may be more sensitive to measuring unhealthy alcohol use at the lower end of the continuum than measures that focus primarily on the consequences of higher severity alcohol use (eg, Cutting Down, Annoyance by Criticism, Guilty Feeling, Eye-Openers [CAGE] Assessment).^64,65^ Utilizing standardized guidelines for identifying risk level and scoring aids (eg, EHR-based assessment of risk level) may help systems in implement comprehensive screening for unhealthy alcohol use.^64,66^

The findings further highlight the importance of evidence-based strategies to integrate screening with treatment and referral for alcohol use in primary care settings in order to engage patients in treatment earlier in the continuum of unhealthy alcohol use as opposed to when advanced consequences have presented.^67,68^ Potential strategies include increased training and certification of primary care providers and staff to address unhealthy alcohol use and addressing shortages in the behavioral health workforce that have hindered systems from meeting patient demand for substance use treatment.^69-71^ Telehealth treatment for alcohol use may serve as a potential strategy for making treatment for unhealthy alcohol use more accessible and desirable, particularly in rural locations and other settings with limited access to sources of care.^72-75^ There is evidence that mobile health interventions can improve alcohol-related outcomes, including decreases in alcohol consumption, binge drinking, and alcohol-related injuries.^76,77^ More evidence is needed to identify features of design and content that may be most effective, desirable, and feasible to disseminate and promote uptake.

Among individuals who face barriers to seeking more traditional sources of treatment or may not require medically-assisted treatment, community-based sources of help such as 12-step programs and support groups can be leveraged as cost-free, accessible, and effective sources of help for unhealthy alcohol use.^78,79^ Additionally, prior research has identified a prominent role for religious organizations in the provision of education, counseling, referral to treatment, encouragement to promote ongoing engagement in treatment, and family support.^80,81^ Further research is required to assess the types of training and services that may be most effective in supportive religious organizations in this role.^81,82^

### Conclusions

This study highlighted factors associated with receipt of help across a broader continuum of alcohol use. Disparities among racial and ethnic minoritized groups in receipt of help and low uptake of treatment underscore the need for interventions to promote awareness, access, and quality of treatment for unhealthy alcohol use.

## Supplemental Material

sj-docx-1-sat-10.1177_29768357241301990 – Supplemental material for Factors Related to Receipt of Help for Alcohol Use: Extending the Focus of Treatment to the Continuum of Unhealthy Alcohol Use Supplemental material, sj-docx-1-sat-10.1177_29768357241301990 for Factors Related to Receipt of Help for Alcohol Use: Extending the Focus of Treatment to the Continuum of Unhealthy Alcohol Use by Lina Tieu, Nadereh Pourat, Elizabeth Bromley, Rajat Simhan, Roshan Bastani and Beth Glenn in Substance Use: Research and Treatment
