# Supplementary material for: Factors Related to Receipt of Help for Alcohol Use: Extending the Focus of Treatment to the Continuum of Unhealthy Alcohol Use
Source: Subst Use. 2024 Nov 25;18:29768357241301990. doi: 10.1177/29768357241301990 (PMC11587183; doi:10.1177/29768357241301990)
Supplement: sj-docx-1-sat-10.1177_29768357241301990 – Supplemental material for Factors Related to Receipt of Help for Alcohol Use: Extending the Focus of Treatment to the Continuum of Unhealthy Alcohol Use [file sj-docx-1-sat-10.1177_29768357241301990.docx]

| Supplementary Tables Supplementary Table 1. Alcohol Consumption and Experiences among Adults with Past Year Unhealthy Alcohol Use, by Drinking Pattern, Unweighted Sample (n=6,467) | | | | |
| --- | --- | --- | --- | --- |
| **Characteristic** | **Exceeding guidelines** | **Binge drinking** | **Heavy alcohol use** | **Total** |
|  | **N=4,814 (74%)** | **N=870 (13%)** | **N=783 (12%)** | **N=6467** |
| **Sex** |  |  |  |  |
| Female | 2,100 (43.6) | 340 (39.1) | 296 (37.8) | 2,736 (42.3) |
| Male | 2,714 (56.4) | 530 (60.9) | 487 (62.2) | 3,751 (57.7) |
| **Age** |  |  |  |  |
| 18 to 34 | 2,121 (44.1) | 487 (56.0) | 336 (42.9) | 2,944 (45.5) |
| 35 to 64 | 2,357 (49.0) | 360 (41.4) | 425 (54.3) | 3,142 (48.6) |
| 65 or over | 336 (7.0) | 23 (2.6) | 22 (2.8) | 381 (5.9) |
| **Race/Ethnicity** |  |  |  |  |
| Hispanic, any race | 973 (20.2) | 222 (25.5) | 135 (17.2) | 1,330 (20.6) |
| Black, non-Hispanic | 1,145 (23.8) | 179 (20.6) | 256 (32.7) | 1,580 (24.4) |
| White, non-Hispanic | 2,481 (51.5) | 418 (48.1) | 353 (45.1) | 3,252 (50.3) |
| Asian/Native Hawaiian/Other Pacific Islander, non-Hispanic | 143 (3.0) | 34 (3.9) | 19 (2.4) | 196 (3.0) |
| American Indian/Alaska Native, non-Hispanic | 72 (1.5) | 17 (2.0) | 20 (2.6) | 109 (1.7) |
| **Education** |  |  |  |  |
| Less than high school | 653 (13.6) | 144 (16.6) | 174 (22.2) | 971 (15.0) |
| High school or GED | 1,344 (27.9) | 237 (27.2) | 285 (36.4) | 1,866 (28.9) |
| Some college | 1,225 (25.5) | 261 (30.0) | 171 (21.8) | 1,657 (25.6) |
| College or associate degree | 1,141 (23.7) | 182 (20.9) | 126 (16.1) | 1,449 (22.4) |
| More than college | 451 (9.4) | 46 (5.3) | 27 (3.5) | 524 (8.1) |
| **Nativity** |  |  |  |  |
| Born in United States | 4,229 (87.9) | 746 (85.8) | 731 (93.4) | 5,706 (88.2) |
| Not born in United States | 582 (12.1) | 124 (14.2) | 52 (6.6) | 758 (11.7) |
| **English Proficiency** |  |  |  |  |
| Speaks English well or very well | 4,566 (96.2) | 819 (95.3) | 759 (98.3) | 6,144 (96.3) |
| Speaks English poorly or very poorly | 182 (3.8) | 40 (4.7) | 13 (1.7) | 235 (3.7) |
| **Income** |  |  |  |  |
| <$20,000 | 1,277 (26.5) | 278 (32.0) | 317 (40.5) | 1,872 (29.0) |
| $20,000 to <40,000 | 1,256 (26.1) | 256 (29.4) | 223 (28.5) | 1,735 (26.8) |
| *Table continued from previous page* |  |  |  |  |
| $40,000 to <70,000 | 1,092 (22.7) | 188 (21.6) | 159 (20.3) | 1,439 (22.3) |
| $70,000 to <100,000 | 564 (11.7) | 74 (8.5) | 53 (6.8) | 691 (10.7) |
| $100,000 or more | 625 (13.0) | 74 (8.5) | 31 (4.0) | 730 (11.3) |
| **Insurance Status** |  |  |  |  |
| No Insurance | 1,238 (26.3) | 284 (33.4) | 273 (35.9) | 1795 (28.4) |
| Any Medicaid | 638 (13.6) | 123 (14.5) | 133 (17.5) | 894 (14.2) |
| Any Private, non-Medicaid | 2,318 (49.3) | 380 (44.7) | 265 (34.8) | 2,963 (46.9) |
| Other Insurance, non-Medicaid, non-Private | 512 (10.9) | 64 (7.5) | 90 (11.8) | 666 (10.5) |
| **Medical Conditions, Last 12 months** |  |  |  |  |
| Any Liver Disease (cirrhosis, liver cancer, other liver disease) | 63 (1.3) | 7 (0.8) | 26 (3.3) | 96 (1.5) |
| Any Non-Liver Cancer | 114 (2.4) | 12 (1.4) | 15 (1.9) | 141 (2.2) |
| **Acute Healthcare Utilization (ED Visit or Hospitalization), Last 12 Months** | 1,166 (24.4) | 239 (28.6) | 218 (28.2) | 1623 (25.3) |
| **Regular Non-Alcohol Substance Use, Last 12 Months** |  |  |  |  |
| Marijuana | 846 (17.6) | 217 (24.9) | 237 (30.3) | 1,300 (20.1) |
| Any Non-Marijuana Substance | 358 (7.4) | 96 (11.0) | 139 (17.8) | 593 (9.2) |
| **Alcohol Use Disorder, Last 12 Months** | 2,272 (47.2) | 544 (62.5) | 610 (77.9) | 3,418 (52.8) |
| None (0 to 1 alcohol-related problems) | 2,545 (52.9) | 328 (37.7) | 176 (22.5) | 3,049 (47.2) |
| Mild (2 to 3 alcohol-related problems) | 1,086 (22.6) | 193 (22.2) | 112 (14.3) | 1,391 (21.5) |
| Moderate (4 to 5 alcohol-related problems) | 582 (12.1) | 143 (16.4) | 135 (17.2) | 860 (13.3) |
| Severe (≥ 6 alcohol-related problems) | 601 (12.5) | 206 (23.7) | 360 (46.0) | 1,167 (18.0) |
| **Received Help for Alcohol Use, Last 12 Months** |  |  |  |  |
| Sought help for drinking (from any source) | 203 (4.2) | 59 (6.8) | 128 (16.4) | 390 (6.0) |
| Perceived need, but did not seek help | 133 (2.8) | 38 (4.4) | 70 (8.9) | 241 (3.7) |
| Did not perceive need for or seek help | 4,478 (93.0) | 773 (88.9) | 585 (74.7) | 5,836 (90.2) |
| **Source of Help for Alcohol Use, Last 12 Months** |  |  |  |  |
| Alcoholics/Narcotics/Cocaine Anonymous or 12-Step Meeting | 122 (2.5) | 36 (4.1) | 81 (10.3) | 239 (3.7) |
| *Table continued from previous page* |  |  |  |  |
| Private physician, psychiatrist, psychologist, social worker, other professional | 84 (1.7) | 24 (2.8) | 59 (7.5) | 167 (2.6) |
| Outpatient clinic, including outreach and day/partial patient program | 49 (1.0) | 19 (2.2) | 39 (5.0) | 107 (1.7) |
| Alcohol/drug rehabilitation program | 39 (0.8) | 16 (1.8) | 31 (4.0) | 86 (1.3) |
| Alcohol/drug detoxification ward/clinic | 27 (0.6) | 13 (1.5) | 37 (4.7) | 77 (1.2) |
| Family Services or other social services agency | 31 (0.6) | 9 (1.0) | 31 (4.0) | 71 (1.1) |
| Emergency room | 22 (0.5) | 9 (1.0) | 36 (4.6) | 67 (1.0) |
| Clergyman, priest, or rabbi | 26 (0.5) | 7 (0.8) | 21 (2.7) | 54 (0.8) |
| Inpatient alcohol/drug ward/clinic | 18 (0.4) | 9 (1.0) | 24 (3.1) | 51 (0.8) |
| Halfway house/therapeutic community | 11 (0.2) | 5 (0.6) | 9 (1.2) | 25 (0.4) |
| Crisis center | 3 (0.1) | 1 (0.1) | 10 (1.3) | 14 (0.2) |
| Employee assistance program | 9 (0.2) | 2 (0.2) | 3 (0.4) | 14 (0.2) |
| Other agency or professional | 12 (0.3) | 3 (0.3) | 6 (0.8) | 21 (0.3) |

GED: General Educational Development, ED: Emergency Department

| Supplementary Table 2. Alcohol Consumption and Experiences among Adults with Past Year Unhealthy Alcohol Use, by Treatment Utilization, Unweighted Sample (n=6,467) | | | | |
| --- | --- | --- | --- | --- |
| **Characteristic, n (row %)** | **Did not receive help** | **Received help** | **Total Who Exceeded Guidelines** | **p-value** |
|  | **N=5,836** | **N=390** | **N=6,467** |  |
| **Sex** |  |  |  | 0.045 |
| Female | 2,590 (95.7) | 146 (5.3) | 2,736 |  |
| Male | 3,487 (93.5) | 244 (6.5) | 3,731 |  |
| **Age** |  |  |  | <.001 |
| 18 to 34 | 2,794 (94.9) | 150 (5.1) | 2,944 |  |
| 35 to 64 | 2,909 (92.6) | 233 (7.4) | 3,142 |  |
| 65 or over | 374 (98.2) | 7 (1.8) | 381 |  |
| **Race/Ethnicity** |  |  |  | 0.001 |
| Hispanic, any race | 1,259 (94.7) | 71 (5.3) | 1,330 |  |
| Black, non-Hispanic | 1,493 (94.5) | 87 (5.5) | 1,580 |  |
| White, non-Hispanic | 3,041 (93.5) | 211 (6.5) | 3,252 |  |
| Asian/Native Hawaiian/Other Pacific Islander, non-Hispanic | 190 (96.9) | 6 (3.1) | 196 |  |
| American Indian/Alaska Native, non-Hispanic | 94 (86.2) | 15 (13.8) | 109 |  |
| **Education** |  |  |  | 0.543 |
| Less than high school | 909 (93.6) | 62 (6.4) | 971 |  |
| High school or GED | 1,745 (93.5) | 121 (6.5) | 1,866 |  |
| Some college | 1,554 (93.8) | 103 (6.2) | 1,657 |  |
| College or associate degree | 1,374 (94.8) | 75 (5.2) | 1,449 |  |
| More than college | 495 (94.5) | 29 (5.5) | 524 |  |
| **Nativity** |  |  |  | 0.003 |
| Born in United States | 5,341 (93.6) | 365 (6.4) | 5,706 |  |
| Not born in United States | 733 (96.7) | 25 (3.3) | 758 |  |
| **English Proficiency** |  |  |  |  |
| Speaks English well or very well | 5,766 (93.9) | 378 (6.2) | 6,144 | 0.083 |
| Speaks English poorly or very poorly | 227 (93.9) | 8 (3.4) | 235 |  |
| **Income** |  |  |  | <.001 |
| <$20,000 | 1,694 (90.5) | 178 (9.5) | 1,872 |  |
| *Table continued from previous page* |  |  |  |  |
| $20,000 to <40,000 | 1,640 (94.5) | 95 (5.5) | 1,735 |  |
| $40,000 to <70,000 | 1,370 (95.2) | 69 (4.8) | 1,439 |  |
| $70,000 to 100,000 | 660 (95.5) | 31 (4.5) | 691 |  |
| $100,000 or more | 713 (97.7) | 17 (2.3) | 730 |  |
| **Insurance Status** |  |  |  | <.001 |
| No Insurance | 1,678 (93.5) | 117 (6.5) | 1,795 |  |
| Any Medicaid | 797 (89.2) | 97 (10.9) | 894 |  |
| Any Private, non-Medicaid | 2,855 (96.4) | 108 (3.6) | 2,963 |  |
| Other Insurance, non-Medicaid, non-Private | 612 (91.9) | 54 (8.1) | 666 |  |
| **Medical Conditions, Last 12 months** |  |  |  |  |
| Any Liver Disease (cirrhosis, liver cancer, other liver disease) | 52 (54.2) | 44 (45.8) | 96 | <.001 |
| Any Non-Liver Cancer | 128 (90.8) | 13 (9.2) | 141 | 0.171 |
| **Acute Healthcare Utilization (ED Visit or Hospitalization), Last 12 Months** | 1,595 (89.8) | 181 (10.2) | 1,776 | <.001 |
| **Regular Non-Alcohol Substance Use, Last 12 Months** |  |  |  |  |
| Marijuana | 1,180 (90.8) | 120 (9.2) | 1,300 | <.001 |
| Any Non-Marijuana Substance | 496 (83.6) | 97 (16.4) | 593 | <.001 |
| **Alcohol Use Disorder, Last 12 Months** |  |  |  | <.001 |
| None (0-1 alcohol-related problems) | 3,008 (98.7) | 41 (1.4) | 3,049 |  |
| Mild (2 to 3 alcohol-related problems) | 1,348 (96.9) | 43 (3.1) | 1,391 |  |
| Moderate (4 to 5 alcohol-related problems) | 809 (94.1) | 51 (5.9) | 860 |  |
| Severe (≥ 6 alcohol-related problems) | 912 (78.2) | 255 (21.9) | 1,167 |  |

GED: General Educational Development, ED: Emergency Department

| Supplementary Table 3. Bivariate Results, Unweighted Sample | | | |
| --- | --- | --- | --- |
| **Characteristic, OR (95% CI)** | **Did not receive help** | **Received help** | **p-value** |
|  | **N=5,836** | **N=390** |  |
| **Unhealthy Alcohol Use, Past 12 Months** |  |  | <.001 |
| Exceeding moderate alcohol use | ref | ref |  |
| Binge drinking | ref | 1.65 (1.22-2.23) |  |
| Heavy alcohol use | ref | 4.44 (3.51-5.62) |  |
| **Age** |  |  | <.001 |
| 18-34 | ref | ref |  |
| 35-64 | ref | 1.49 (1.21-1.84) |  |
| 65+ | ref | 0.35 (0.16-0.75) |  |
| **Sex** |  |  | 0.043 |
| Male | ref | ref |  |
| Female | ref | 0.81 (0.65-0.995) |  |
| **Race/Ethnicity** |  |  | 0.004 |
| White, non-Hispanic | ref | ref |  |
| Hispanic, any race | ref | 0.81 (0.62-1.07) |  |
| Black, non-Hispanic | ref | 0.84 (0.65-1.09) |  |
| Asian/Native Hawaiian/Other Pacific Islander, non-Hispanic | ref | 0.46 (0.20-1.04) |  |
| American Indian/Alaska Native, non-Hispanic | ref | 2.30 (1.31-4.04) |  |
| **Education** |  |  | 0.531 |
| Less than high school | ref | ref |  |
| High school or GED | ref | 1.02 (0.74-1.40) |  |
| Some college | ref | 0.97 (0.70-1.35) |  |
| College or associate degree | ref | 0.80 (0.57-1.13) |  |
| More than college | ref | 0.86 (0.55-1.35) |  |
| **Income** |  |  | <.001 |
| <$20,000 | ref | ref |  |
| $20,000 to <40,000 | ref | 0.55 (0.43-0.71) |  |
| $40,000 to <70,000 | ref | 0.48 (0.36-0.64) |  |
| $70,000 to 100,000 | ref | 0.45 (0.30-0.66) |  |
| $100,000 or more | ref | 0.23 (0.14-0.38) |  |
| *Table continued from previous page* |  |  |  |
| **Nativity** |  |  | <.001 |
| Born in United States | ref | ref |  |
| Not born in United States | ref | 0.50 (0.33-0.75) |  |
| **English Proficiency *assumes English-only speakers exempt** |  |  | 0.061 |
| Speaks English Well or Very Well | ref | ref |  |
| Speaks English Poorly or Very Poorly | ref | 0.54 (0.26-1.10) |  |
| **Insurance Status** |  |  | <.001 |
| No Insurance | ref | ref |  |
| Any Medicaid | ref | 1.75 (1.32-2.31) |  |
| Any Private, non-Medicaid | ref | 0.54 (0.41-0.71) |  |
| Other Insurance, non-Medicaid, non-Private | ref | 1.27 (0.90-1.77) |  |
| **Medical Conditions, Last 12 months** (ref: no respective disease) |  |  | <.001 |
| Any Liver Disease (cirrhosis, liver cancer, other liver disease) | ref | 14.84 (9.79-22.50) |  |
| Any non-liver cancer (including breast, oropharyngeal, other cancer) | ref | 1.61 (0.90-2.87) | 0.073 |
| **Acute Healthcare Utilization (ED Visit or Hospitalization), Last 12 months** (ref: no visit) | ref | 2.46 (2.00-3.03) | <.001 |
| **≥ Monthly Non-Alcohol Substance Use Use, Last 12 Months** |  |  |  |
| Marijuana | ref | 1.84 (1.47-2.31) | <.001 |
| Any Non-Marijuana Substance | ref | 3.73 (2.92-4.78) | <.001 |
| **Number of Alcohol-related Problems, Last 12 Months** | ref | 1.49 (1.44-1.54) | <.001 |

GED: General Educational Development, ED: Emergency Department

| Supplementary Table 4. Multivariable Results for Receipt of Help for Unhealthy Alcohol Use, Unweighted Sample | | |
| --- | --- | --- |
| **Characteristic** | **OR (95% CI)** | **p-value** |
| **Unhealthy Alcohol Use, Past 12 Months** |  |  |
| Exceeding moderate alcohol use | ref |  |
| Binge drinking | 1.01 (0.71-1.44) | 0.949 |
| Heavy alcohol use | **1.43 (1.06-1.93)** | **0.019** |
| **Age** |  |  |
| 18-34 | ref |  |
| 35-64 | **1.56 (1.21-2.01)** | **0.001** |
| 65+ | 0.68 (0.29-1.6) | 0.375 |
| **Sex** |  |  |
| Male | ref |  |
| Female | **0.73 (0.56-0.95)** | **0.017** |
| **Race/Ethnicity** |  |  |
| White, non-Hispanic | ref |  |
| Hispanic, any race | 0.89 (0.62-1.26) | 0.508 |
| Black, non-Hispanic | **0.67 (0.49-0.91)** | **0.012** |
| Asian/Native Hawaiian/Other Pacific Islander, non-Hispanic | 0.67 (0.20-2.29) | 0.524 |
| American Indian/Alaska Native, non-Hispanic | 1.07 (0.51-2.23) | 0.866 |
| **Education** |  |  |
| Less than high school | ref |  |
| High school or GED | 1.39 (0.94-2.04) | 0.096 |
| Some college | **1.65 (1.09-2.47)** | **0.017** |
| College or associate degree | **1.84 (1.2-2.84)** | **0.006** |
| More than college | **2.97 (1.67-5.30)** | **<.001** |
| **Income** |  |  |
| <$20,000 | ref |  |
| $20,000 to <40,000 | 0.8 (0.58-1.09) | 0.150 |
| $40,000 to <70,000 | 0.79 (0.55-1.13) | 0.191 |
| $70,000 to <100,000 | 0.8 (0.49-1.30) | 0.369 |
| $100,000 or more | **0.46 (0.26-0.83)** | **0.010** |
| **Nativity** |  |  |
| Born in United States | ref |  |
| Not born in United States | 0.63 (0.36-1.10) | 0.103 |
| **English Proficiency** |  |  |
| Proficiency | ref |  |
| Limited Proficiency | 1.35 (0.52-3.48) | 0.534 |
| **Insurance Status** |  |  |
| No Insurance | ref |  |
| Any Medicaid | **1.78 (1.25-2.52)** | **0.001** |
| Any Private, non-Medicaid | **0.69 (0.50-0.97)** | **0.031** |
| Other Insurance, non-Medicaid, non-Private | 1.35 (0.90-2.04) | 0.147 |
| **Medical Conditions, Last 12 months** (ref: no respective disease) |  |  |
| Any Liver Disease (cirrhosis, liver cancer, other liver disease) | **6.73 (3.93-11.53)** | **<.001** |
| Any non-liver cancer (including breast, oropharyngeal, other cancer) | 1.09 (0.51-2.33) | 0.829 |
| **Any Acute Healthcare Utilization (ED or Hospitalization), Last 12 months** (ref: no visit) | **1.54 (1.20-1.99)** | **0.001** |
| **≥ Monthly Non-Marijuana Substance Use, Last 12 Months** (ref: < monthly use) | **1.43 (1.04-1.95)** | **0.028** |
| **≥ Monthly Marijuana Use, Last 12 Months** (ref: < monthly use) | 0.89 (0.67-1.19) | 0.440 |
| **Number of Alcohol-related Problems, Last 12 Months** | **1.43 (1.37-1.49)** | **<.001** |

GED: General Educational Development, ED: Emergency Department

Supplementary Table 5. Specification of Variables

| Measure | Construction/Coding | NESARC-III Questions Used in Variable Construction^42^ |
| --- | --- | --- |
| Age | 18-34; 35-64; 65 or older | How old are you as of today? |
| Gender | Female;  Male | What is your sex? |
| Educational attainment | Less than high school;  High school or GED;  Some college;  College or associate degree;  More than college | What is the highest grade or year of school that you completed? |
| Race/ethnicity | Hispanic, any race;  Black, non-Hispanic;  White, non-Hispanic;  Asian/Native Hawaiian/Other Pacific Islander, non-Hispanic;  American Indian/Alaska Native, non-Hispanic | Are you of Hispanic or Latino origin?  Please select 1 or more categories to describe your race. [White, Black or African American, Asian, Native Hawaiian or Other Pacific Islander, American Indian or Alaska Native] |
| Nativity | Born in United States;  Not born in United States | Were you born in the United States? |
| Income | <$20,000; 20,000-<40,000; 40,000-<70,000; 70,000-<100,000; 100,000 or more | Please tell me which category on this card best represents YOUR TOTAL COMBINED HOUSEHOLD income in  the last 12 months. |
| English proficiency | (1) Poorly or very poorly, or (2) Well or very well | How well do you speak English? |
| Health insurance status | (1) No insurance, (2) Any Medicaid, (3) Any Private, non-Medicaid (4) Other Insurance, non-Medicaid, non-Private | At ANY time during the last 12 months were YOU covered by…   1. Medicare? 2. Medicaid or (local name)? 3. TRICARE, CHAMPUS, CHAMPVA, the VA, or other military health care? 4. A private health insurance plan obtained through a current or former employer or union? 5. A private health insurance plan purchased DIRECTLY by you or a relative? 6. A private health insurance plan through state or local government or community program? 7. Any OTHER government or state-sponsored health insurance plan or program? 8. Any OTHER health insurance plan? |
| Liver Disease | Any cirrhosis, liver cancer, or other liver disease | During the last 12 months, did you have…   1. Cirrhosis of the liver? 2. Any other form of liver disease? 3. Liver cancer? |
| Non-liver Cancer | Any non-liver cancer | During the last 12 months, did you have…   1. Any other cancer? |
| Acute Health Care Utilization | Any ED visit or hospitalization, last 12 months | (Not counting hospitalization for delivery of a healthy live born infant,) How many separate times did you stay OR in a hospital overnight or longer in the last 12 months?  During the last 12 months, how many times did you times receive medical care or treatment in a hospital OR emergency room? |
| Marijuana Use | At least monthly use of marijuana | During the last 12 months, about how often did you use marijuana? |
| Use of Non-Marijuana Substances | At least monthly use of any listed drug | During the last 12 months, about how often did you use …   1. Sedatives or Tranquilizers 2. Painkillers 3. Cocaine or Crack 4. Stimulants 5. Club drugs 6. Hallucinogens 7. Inhalants/Solvents 8. Heroin 9. OTHER |
| Alcohol-related problems | Number of alcohol-related problems | See Supplementary Table 6 |
| Alcohol Use Disorder | None: 0-1 alcohol-related problems, Mild: 2-4 problems, Moderate: 4-5 problems, Severe: 6 or more problems | See Supplementary Table 6 |
| Receipt of Help for Alcohol Use | Receipt of help from any source: Alcoholics/Narcotics/Cocaine Anonymous or 12-Step Meeting  Private physician, psychiatrist, psychologist, social worker, other professional  Outpatient clinic, including outreach and day/partial patient program  Alcohol/drug rehabilitation program  Alcohol/drug detoxification ward/clinic  Emergency room  Family Services or other social services agency  Clergyman, priest, or rabbi  Inpatient alcohol/drug ward/clinic  Halfway house/therapeutic community  Crisis center  Employee assistance program  Other agency or professional | Did you go there during the last 12 months ONLY, before the last 12 months ONLY or during both time periods?   1. Alcoholics Anonymous, Narcotics or Cocaine Anonymous meeting, or any 12-step meeting? 2. Family services or other social service agency? 3. Alcohol or drug detoxification ward or clinic? 4. Inpatient ward of a psychiatric or general hospital or community mental health program? 5. Outpatient clinic, including outreach programs and day or partial patient programs? 6. Alcohol or drug rehabilitation program? 7. Emergency room for any reason related to your drinking? 8. Halfway house, including therapeutic communities? 9. Crisis center for any reason related to your drinking? 10. Employee assistance program (EAP)? 11. Clergyman, priest, rabbi or any other religious counselor for any reason related to your drinking? 12. Private physician, psychologist, social worker, or any other professional? 13. Any other agency or professional? |

NESARC-III: National Epidemiologic Survey on Alcohol and Related Conditions-III
